# Adult—Juvenile interactions and temporal niche partitioning between life-stages in a tropical amphibian

**DOI:** 10.1371/journal.pone.0238949

**Published:** 2020-09-14

**Authors:** Diana Székely, Dan Cogălniceanu, Paul Székely, Mathieu Denoël

**Affiliations:** 1 Laboratorio de Ecología Tropical y Servicios Ecosistémicos - EcoSs Lab, Departamento de Ciencias Biológicas, Universidad Técnica Particular de Loja, Loja, Ecuador; 2 Faculty of Natural and Agricultural Sciences, Ovidius University Constanța, Constanța, Romania; 3 Laboratory of Ecology and Conservation of Amphibians (LECA), Freshwater and OCeanic science Unit of reSearch (FOCUS), University of Liège, Liège, Belgium; 4 Asociation Chelonia, București, Romania; University of Fribourg, SWITZERLAND

## Abstract

Divergence in ecological niche offers organisms the opportunity of exploiting different food and habitat resources, scaling down competition and predation both among species, and within different age or size-classes of the same species. In harsh environments, where abiotic factors determine a clustering of resources during short timespans, competition and predation between organisms is likely to be enhanced. This is the case in tropical dry forests, where amphibians have limited opportunities to feed, their activity being restricted to the short rainy season. One way to maximize resource exploitation while avoiding predation risk is by adopting different diel activity patterns. We tested this hypothesis by comparing activity patterns in adults and recently metamorphosed juveniles of Pacific horned frogs (*Ceratophrys stolzmanni*) during field surveys and in an experimental study. Field surveys showed that the adults are strictly nocturnal, whereas freshly metamorphosed juveniles can be found active above ground at all hours, with a peak activity during daytime. The average body condition index of juveniles found active during the night was higher than that of juveniles found active during the day, suggesting that the weaker individuals may be constrained to being active during the day. On the other hand, in a laboratory experiment, juveniles that were visually exposed to adults moved less than those in the absence of adults. Both field and experimental observations indicate a temporal niche divergence between life stages. The results of the experiment offer support to the hypothesis that the juveniles in this species display an inverse activity pattern compared to adults, which can reduce competitive interactions and predation pressure from the larger conspecifics.

## Introduction

The niche concept is essential to our understanding of community ecology [1]. Divergence in resource use as a result of interspecific interaction has been extensively documented in many taxa [2–4], but to a lesser extent for intraspecific interactions [5, 6]. The preferred ecological niche is predicted to offer optimal physiological conditions, maximizing the encounter rate with appropriate prey, while minimizing interactions with both predators and competitors [7]. Individual body size affects many life-history aspects, such as diet, physiology, behaviour, and predation [8–11], so it is expected that different body-size classes will differ in their ecological niche [12–14].

In the case of animals with complex life cycles, many of which have aquatic larvae and terrestrial adults (such as many amphibians), the changes in ecological niche at metamorphosis are obvious [15–17], while changes that take place during the terrestrial stage are largely disregarded. Overall, the best studied ontogenetic niche shifts refer to changes in habitat use [18–20] and diet [21, 22]. The temporal axis of the ecological niche is another dimension of the ecological niche along which species can separate [23]. Temporal partitioning reduces competition for resources [24, 25], and can decrease exposure to predation risks [26]. However, major shifts in diel activity are much less ubiquitous than microhabitat or diet partitioning, especially amongst closely related taxa, and even less so amongst conspecifics, because activity patterns are evolutionarily constrained [27]. In the case of anurans, the vast majority of species avoid diurnal activity in terrestrial stages [28, 29], since nighttime offers protection from desiccation, higher temperatures [30], and some predators [31]. Because of their permeable wet skin used for respiration, amphibians are prone to desiccation, and, since the ratio between surface and volume is higher in smaller animals, juveniles are theoretically more susceptible to dehydration compared to adults [32–34]. As a result, it is expected that juveniles would be more likely to be active during nighttime [35, 36]. Nevertheless, in a small number of species, empirical observations show that the opposite phenomenon takes place, with adults being predominantly nocturnal and juveniles exhibiting diurnal activity [35, 37]. In bufonid species, this is considered an adaptation that allows small juveniles to benefit from warmer conditions, which in turn promotes faster growth [38, 39]. However, at least in some cases, an alternative hypothesis is that the temporal shift in activity allows juveniles to reduce predation risk from larger conspecifics [40], and to avoid competition over the trophic resource [41].

The Pacific horned frog (*Ceratophrys stolzmanni*) is a fossorial amphibian inhabiting the Neotropical seasonally dry forests of coastal Ecuador and Peru [42]. These frogs are active during the rainy season (January–May), reproduce in February–March and juveniles metamorphose after a short larval period, that lasts two–three weeks [43]. Juveniles metamorphose at a relatively large size (approx. 55% of the adult size; [44]), and both juveniles and adults are easy to spot when not buried underground, both during the day and at night. Like other members of the family Ceratophryidae, the Pacific horned frog is anurophagous, being able to swallow large vertebrate prey species [45]. The extremely seasonal environment concentrates the activity of these frogs to a short time period each year, making competition for resources and the agonistic interactions between conspecific individuals likely. We hypothesized that a temporal partitioning between adult and juvenile life-stages occurs in the population, which would be adaptive by reducing competition and predation risk (i.e. cannibalism). The avoidance of adults by juveniles because of predation risk (the cannibalistic hypothesis) was specifically tested in a laboratory experiment, in which we tested if adult presence impacts juvenile activity, expecting that visual exposure to a large frog should reduce movement in juveniles. Additionally, we expected differences in body condition between juveniles foraging during nighttime compared to those active during daytime, under the hypothesis that juveniles with a lower body mass are constrained to activity under less favourable conditions (dryness and higher temperatures) to avoid interaction with adults.

## Materials and methods

Research permit for the study was issued by Ministerio del Ambiente del Ecuador (MAE-DNB-CM-2015-0016, granted to Universidad Técnica Particular de Loja). All applicable institutional and/or national guidelines for the care and use of animals were followed. The study was approved by the Universidad Técnica Particular de Loja Ethics Committee (UTPL-CBEA-2016-001).

### Field sampling

**Fig 1:**
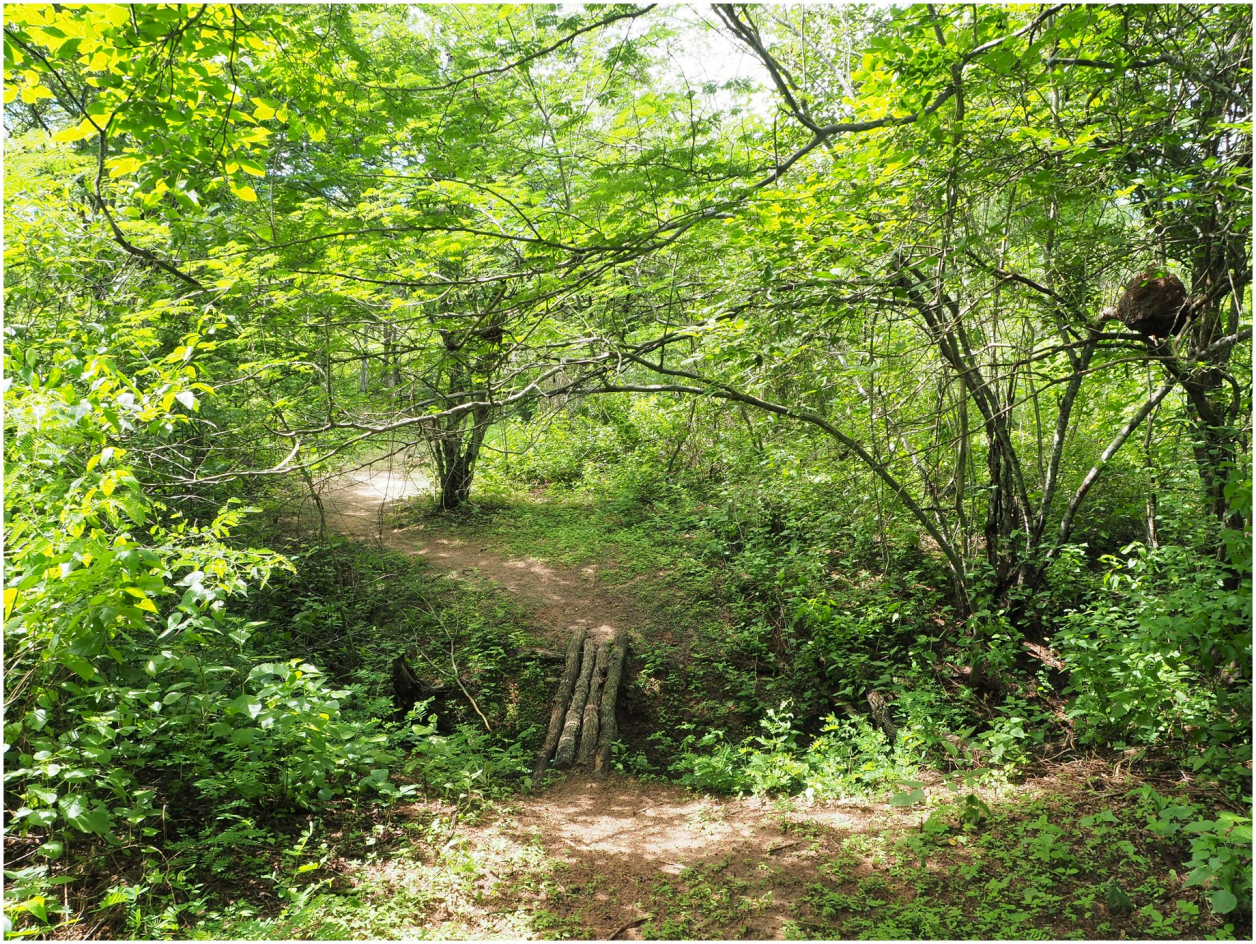
Forest trail at the study site (Arenillas Ecological reserve, Ecuador).

The study was carried out in Arenillas Ecological Reserve, southern Ecuador (03°34'S; 80°08'E, 30 m a.s.l.), during the rainy season (21 January–18 March, 2016), along a 2.5 km transect, consisting of narrow forest trails in the centre of the reserve (Fig 1), with low human impact and a relatively homogenous vegetation [46]. Where the path was travelled twice (going and returning), animals were counted only during the first pass. At this location and for the duration of the study, sunrise time varied between 06:22 and 06:24, while sunset was between 18:31 and 18:40. As a result, we assigned surveys carried out between 9:00 and 18:59 as diurnal, and between 19:00 and 7:00 as nocturnal. Field observations started at various hours (17:00, 19:00, 21:00, 23:00, 01:00, 03:00, 05:00), with one transect lasting 2h during the whole study interval. For a week after the onset of metamorphosis of horned frogs (11–18 March), each night transect was doubled by another transect during the day, starting at either 09:00, 11:00, 13:00, 15:00, or 17:00 (S1 Table). Only individuals at Gosner stage 46 (tail completely resorbed; [47]) or older were counted. Frogs were considered adults if having snout-vent length (SVL) over 50 mm, and juveniles if under 45 mm [44]. Measured juveniles had a mean SVL ± S.E. of 29.9 ± 0.2 (range: 23.2–42.9 mm, *n* = 286), while adult SVL varied between 50.3 and 77.8 mm (61.5 ± 0.17, *n* = 564). Air temperature and relative humidity were measured on location, using an EL-21CFR-2-LCD-UK data logger (Lascar Electronics, Wiltshire, UK) positioned 2 m above ground, in the shade, set to record the parameters each minute.

Of the encountered juveniles, 20 randomly-chosen individuals were measured during each transect whenever available (SVL with a Dial-Max calliper of 0.1 mm precision, body mass—BM with a My Weigh 300Z portable scale of 0.1 g precision). Measurements were taken *in situ*, and the individuals were immediately released at the capture point. These values served to calculate the body condition index (BCI) of individuals as the residuals of the regression of lnBM (body mass) on lnSVL [48], which is a good estimate of energy storage in amphibians [49].

### Laboratory experiments

On 15–17 March 2016, we conducted an experiment, testing for behavioural interaction between adults and juveniles. We collected 15 adults and 30 freshly metamorphosed juveniles (Gosner stage 46) from the reserve (about 500 m from the location of the transect). All individuals were kept individually in plastic enclosures (50 x 37 cm, 30 cm height for adults; 21 x 15cm, 12 cm height for juveniles), with a layer of 6 cm humid soil (same as in their capture site) to acclimate for approximately 24 h previously to being used in the experiments. The laboratory was situated on site, with natural conditions (daylight duration, temperature, humidity). Experimental enclosures consisted of 50 x 37 cm, 30 cm height, plastic boxes, split into two arenas of equal surface by a transparent glass, and monitored from above with a Logitech HD Pro C920 webcam (resolution: 1920 x 1080 pixels), situated 150 cm above the enclosures, connected to a laptop. To improve visibility during the experiments, lighting was supplemented by ExoTerra Infrared light bulbs (50W, 800 nm) that do not affect the activity of frogs [50]. Using Chronolapse 1.0.7 software (Green 2008, https://code.google.com/p/chronolapse), time-lapse photos were taken every 5 sec, for 1 h duration, starting 10 min after the introduction of experimental individuals.

**Fig 2:**
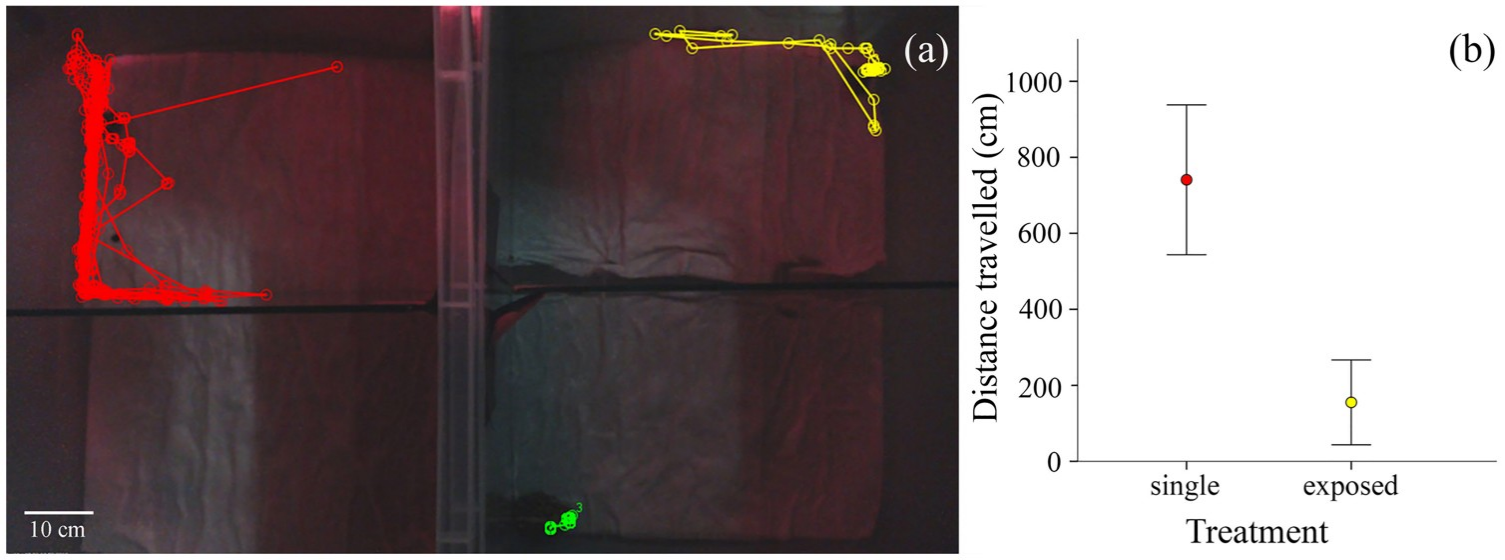
Influence of adult presence on juvenile activity: (a) examples of tracks (i.e. individual movements) of the juveniles in the experimental design. The left and right enclosures correspond to the single (i.e. no adult) and the exposed (i.e. adult visible) treatments, respectively. Each enclosure consisted of two arenas separated by transparent glass. The coloured paths indicate the distance travelled by individuals during 1 h recording (red and yellow: juveniles; green: adult); (b) distance travelled (mean ± S.E.) in the experimental enclosure during 1 h by juveniles, without and with the presence of an adult.

Juveniles were assigned randomly to one of two treatments (absence vs. presence of an adult; *n* = 15 juveniles for each treatment), with no difference between them in SVL (Student's *t*-test, *t*_28_ = -0.524, *p* = 0.605) or BCI (*t*_28_ = -1.289, *p* = 0.208). Two open-top experimental enclosures (randomized position), lined with moist tissue paper, separated by an opaque wall, were monitored at the same time: (1) one with a juvenile in one arena and an adult on the other side of the glass, in the other arena (Fig 2a, right enclosure), and (2) the second identical to the first, but without the adult (Fig 2a, left enclosure). The enclosures were thoroughly washed and sun-dried between trials, in an attempt to remove any possible chemical cues from previous individuals. Each individual was used only once and released after the experiment at the capture site.

Images (720 per replicate, i.e. 10,800 in total) were processed using ImageJ 1.46r software (U.S. National Institute of Health) along with the MTrackJ 1.5.0 plugin [51], that allows tracking movement of study animal, through manual input of position in the enclosure on each individual image [52]. We used the total distance travelled, in cm, during 1h, as a measure of individual activity.

### Statistical analysis

All analyses were carried out with SPSS software, version 21.0 (IBM Corp., Armonk, NY), with a significance level of 0.05 and two-tailed tests. Parameters that after QQ-plot inspection were considered not normally distributed (i.e. the number of individuals encountered during surveys and distance travelled in experimental trials) were ln-transformed prior to analysis. To determine the temporal niche partitioning between adults and juveniles, we used the Czekanowski index of overlap [53]: $\alpha =1-0.5(\sum_{i=1}^{n}\left|p_{i.ad}-p_{i.juv}\right|)$, where *p*_*i*.*ad*_ is the proportion of adults active during the 2-h interval *i*, and *p*_*i*.*juv*_ is the proportion of juveniles active during the same interval *i*. The values of the index range between 0 (i.e. full partitioning between time intervals used), and 1 (i.e. full overlap in temporal niche use). We used Student's *t*-tests to determine significant differences in the number of juveniles counted during transects, their body condition, and the meteorological conditions (temperature and humidity, recorded at the middle time of the survey) during diurnal vs. nocturnal surveys. We used linear regressions to test for the effect of SVL and BCI on the distance travelled by juveniles in the laboratory experiment. The distances travelled by juveniles in absence versus presence of an adult during the experimental tests were compared with a Student’s *t*-test. Additionally, we tested through linear regressions the predictive value of two traits of the adults on exposed juvenile distance travelled: adult activity (also measured as distance travelled), and size (measured as SVL). We included both parameters in the model since adult activity and SVL were not correlated (*r* = 0.14, *F*_1,13_ = 0.275, *p* = 0.609).

## Results

### Field survey

**Fig 3:**
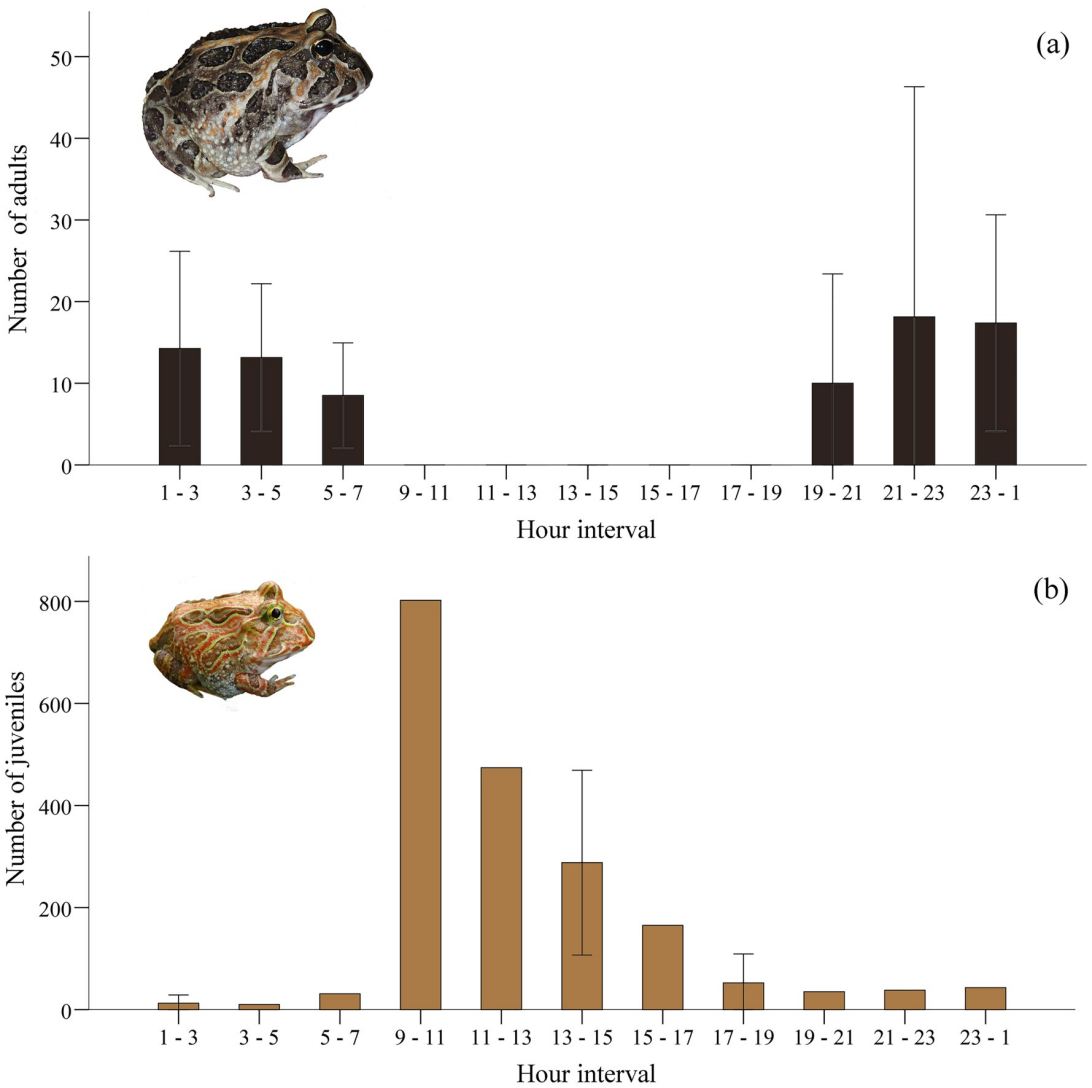
Effect of the time of day on number of encountered horned frogs, according to life-stage: (a) adults (dark brown); (b) juveniles (light brown). Surveys between 7:00–19:00 considered diurnal, and those between 19:00–7:00 nocturnal. Bars: mean; whiskers: S.E.

A total of 621 adults and 2,356 juveniles were encountered during the study period. We found a strong difference in the time of day when the two life-stages were active above ground, with a value of the Czekanowski index of temporal overlap of 0.074. Adults were exclusively nocturnal, with toads coming out of the ground not sooner than 19:04 and hiding in their burrows before 06:15, and variable numbers being active throughout the night, particularly between 23:00 and 5:00 (Fig 3a). Juveniles could be encountered at all hours, but the occurrence rate differed according to the time of day, with a peak of activity between 9:00 and 11:00 (Fig 3b).

During the week following the metamorphosis of juveniles (11–18 March), climatic conditions differed between the nocturnal transects and diurnal transects (Student's *t*-test, temperature: *t*_8.3_ = 5.404, *p* = 0.001; relative humidity: *t*_7.58_ = - 4.218, *p* = 0.003): average temperature was higher (mean ± S.E. = 31.3 ± 0.8 °C) and relative humidity lower (mean ± S.E. = 73.1 ± 3.2%) during the day compared to during the night (temperature 26.5 ± 0.3 °C; relative humidity 86.7 ± 0.6%; S1 Fig).

**Fig 4:**
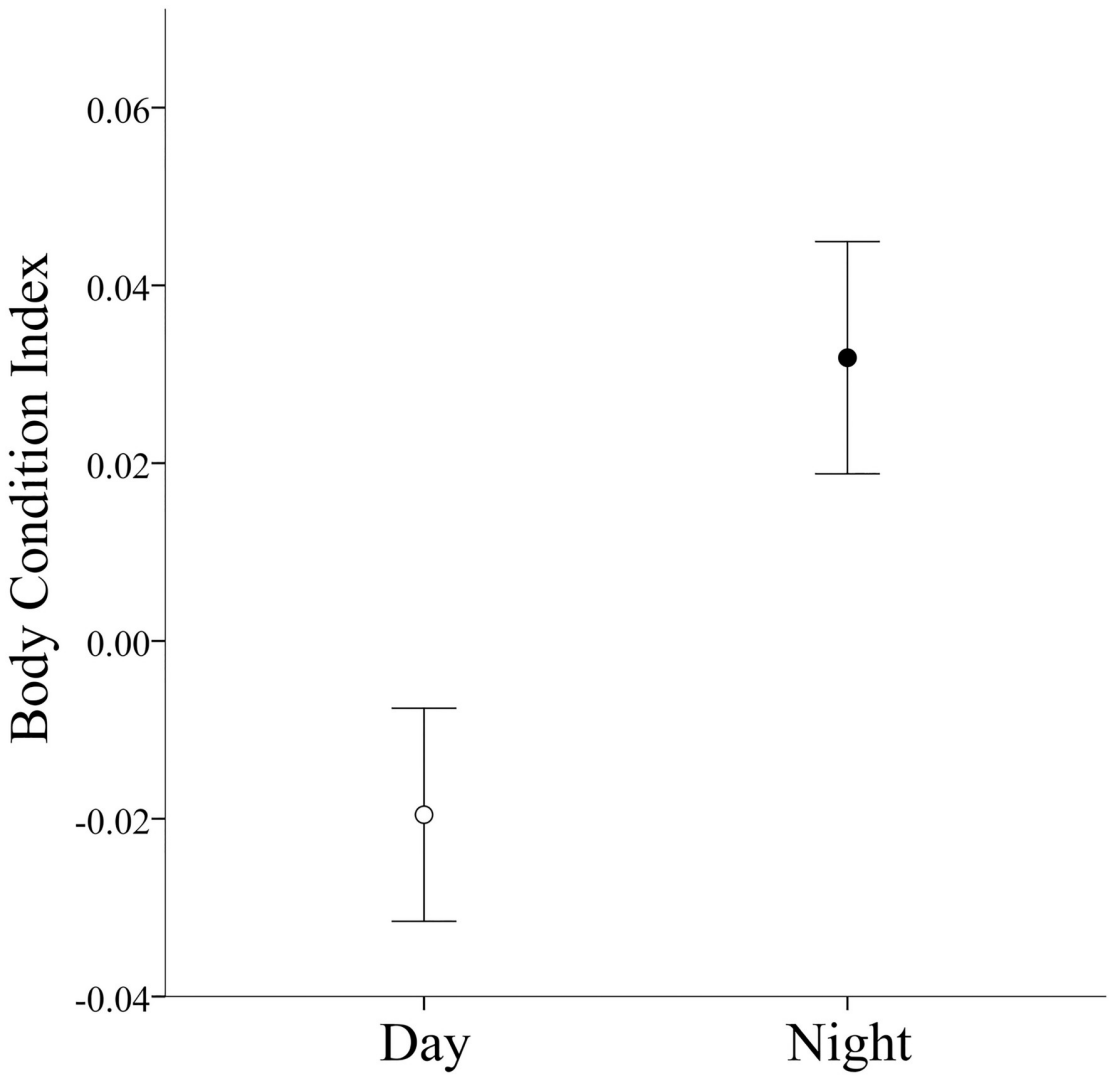
Differences in body condition (mean ± S.E.) between juvenile Pacific horned frogs active during the day (*n* = 176) compared to those active during the night (*n* = 108).

The number of encountered juveniles was larger during diurnal surveys (mean ± S.E. = 272 ± 96 / survey) than at night (26 ± 6 / survey; Student's *t*-test, *t*_13_ = 2.749, *p* = 0.017). Juvenile individuals that were found active during the night were in significantly better body condition than those found active during the day (average BCI ± S.E.: night = 0.03 ± 0.01, day = -0.019 ± 0.01; Student's *t*-test, *n*_day_ = 176, *n*_night_ = 108, *t*_282_ = 2.793, *p* = 0.006; Fig 4).

### Behavioural experiment

Juveniles that were visually exposed to the presence of an adult moved less during the 1h monitored interval (mean ± S.E. = 155.1 ± 111.7 cm) than in the absence of adults (740.8 ± 197.2 cm; Student's *t*-test, *t*_28_ = -3.99, *p* < 0.001 (Fig 2b). The distance travelled by the juveniles exposed to the sight of an adult was not influenced by the size of the adult (*r*^2^ = 0.015, *F*_1,13_ = 0.195, *p* = 0.666), nor by the activity of the adult (*r*^2^ = 0.061, *F*_1,13_ = 0.841, *p* = 0.376). Overall, neither the size of the juvenile (*r*^2^ = 0, *F*_1,28_ = 0.003, *p* = 0.954), nor its body condition (*r*^2^ = 0.02, *F*_1,28_ = 0.558, *p* = 0.461) affected its mobility (S2 Fig). However, the distance travelled was positively correlated with juvenile BCI in the absence of an adult (*r*^2^ = 0.268, *F*_1,13_ = 4.753, *p* = 0.048).

## Discussion

The study shows that, in their post-metamorphic, terrestrial stages, Pacific horned frogs display an ontogenetic change in the temporal niche used, with adults being strictly nocturnal, while juveniles are mainly active during daytime. Our results emphasize the fact that important changes in the ecological niche are not limited to the metamorphic event, and shed new insights on intraspecific temporal resource partitioning, one of the least studied aspects of community structuring [27]. The results contrast with most previous research that showed amphibians to be conservative in terms of preferred diel activity throughout their terrestrial lives [28, 54] because of the strong dependence of their activity on climatic factors [34]. Indeed, in the majority of studied fossorial amphibians both juveniles and adults are nocturnal [35, 55–57]; alternatively, in a few species, juvenile dispersal can occur during the crepuscular hours, juveniles moving either early in the morning or shortly after sunset, and only after heavy rains [58]. However, since the observations here presented are limited to only one activity season, a better understanding of the impact of climatic factors might result from repeated observations spanning several years.

In some systems, biological interactions are more important than environmental pressures in determining activity patterns [40, 59, 60]. We interpreted the lower activity levels of juveniles in the presence of adults as an antipredator mechanism [61, 62], which can efficiently mitigate predation risks associated with morphology, in this case small size [63]. The fact that *Ceratophrys* juveniles modified their behaviour in response to the presence of conspecifics supports the hypothesis that juveniles might avoid intraspecific interactions by diverging from adult typical activity time-frame in their most vulnerable stage. These results extend the observations made by Schalk and Fitzgerald [64] regarding a related species, *Ceratophrys cranwelli*, that inhabits similar seasonally dry environments and is frequently anurophagous [65]. In this species there was a strong divergence in micro-habitat use, with juveniles hunting in drier areas and avoiding the optimal humid habitats close to ephemeral ponds that were dominated by large individuals [64]. In our study, we did not analyse the spatial distribution of life-stages; however, we found both juveniles and adults on the same portions of the monitored transect. Altogether, our results and those of Schalk & Fitzgerald [64] suggest that juveniles can employ both spatial and temporal strategies to avoid adults.

Being gape-limited predators, the maximum size of ingested prey increases along with individual size [66], and large individuals have a more diverse diet through access to larger prey items [67]. However, large individuals do not disregard small prey organisms [45], a typical behaviour found in other amphibians [65, 68]. As such, we could expect a significant dietary overlap between age-classes. Consequently, in addition to lowering predation risk, the observed partitioning along the temporal niche axis should be effective in relaxing competition between juveniles and adults, reducing the overlap in resource use and the agonistic interactions between age-classes [27].

Our field observations showed that the nighttime niche is dominated by adults, along with a non-random subset of juveniles with higher body condition. In this context, it seems plausible that the less fit juveniles might be constrained to diurnal activity in order to improve their foraging efficiency by taking advantage of food sources that are less exploited during the day [69, 70], thus avoiding competition and predation risks from both adults and the better fit juveniles. The lower body condition found in diurnal juveniles as compared to nocturnal juveniles suggests a lower payoff of the diurnal behaviour. This situation may be considered as a best of a bad lot tactic [71]. Therefore, part of the juveniles might “choose” nocturnal activity, which is more energetically profitable and involves a lower hydric stress, but also implies more risks; their success depending on the density of adults.

No data regarding diel variation in prey availability is available for the study site. However, in other seasonally dry tropical environments, the ground-level insect availability changes little between daytime and nighttime, both in terms of species composition and abundance [72, 73]. Further studies on differential distribution and availability of prey and energy acquisition according to diel patterns, as well as preferred prey types in relation to developmental stage, might test if variations in prey availability make diurnal activity beneficial for early-stages terrestrial horned frogs.

Along with competition and predation risk from conspecific adults, there are several other, non-exclusive, determinants that could explain the observed dichotomic pattern of activity between the life-stages, such as differences in physiological tolerance between life stages, mainly to desiccation risk, or susceptibility to predation (other than cannibalism), compared to adults. Since in juveniles the ratio between surface and volume is smaller than in adults, resulting in a higher desiccation risk, theory predicts that the preference for nocturnal activity should be accentuated in juveniles, especially in hot climates [32]. However, amongst anurans, some bufonid species can be described as heliophilic in their early post-metamorphic stages, benefiting from the increased temperatures, as they intensify growth rates [74]. In such species, juveniles are active during daytime and have a higher tolerance for critically high temperatures compared to the nocturnal adults [37]. To mitigate the risk of dehydration and overheating, they can remain clustered in humid habitats in close vicinity to their emergence ponds [39, 75], and have thinner, less cornified skin, allowing them to rehydrate more quickly [37]. Additionally, most diurnally active amphibian species benefit from protection from predators through toxic skin secretions. It is for instance typically the case of bufonids [76], dendrobatids [77], and salamandrids [31, 78].

In comparison to some other anurans, horned frogs (genus *Ceratophrys*) are poorly equipped for cutaneous chemical defence [79], and rely mostly on camouflage [80] and behavioural strategies [81] to avoid predation. Further testing of other hypotheses will improve our understanding of the mechanisms that structure the activity patterns within populations in extremely seasonal environments, such as size-dependent susceptibility to predation or specific physiological or morphological adaptations that allow Pacific horned frog juveniles to be active during daytime, as well as individual variations (behavioural types).

## Supporting information

S1 Fig (a) Active Pacific horned frog in its habitat; (b) climate conditions during surveys on 10–18 March 2016, according to the time of day (red line—temperature, blue line—relative humidity) and the number of juveniles encountered during the surveys (during the day—white bars, during the night—full bars). (TIF)

S2 Fig Distance travelled by Pacific horned frog juveniles (*n* = 30), in relation to their Snout-Vent Length (a) and body condition (b). Red dots—single (i.e. no adult), and yellow dots—exposed (i.e. adult visible) treatments, respectively.
(TIF)

S1 Table Schedule of transects and number of encountered Pacific horned frogs, according to their life-stage. (DOCX)

S1 Appendix Raw data supporting data. (XLSX)
