# Supplementary figures and images for: Adult—Juvenile interactions and temporal niche partitioning between life-stages in a tropical amphibian
Source: PLoS One. 2020 Sep 14;15(9):e0238949. doi: 10.1371/journal.pone.0238949 (PMC7489520; doi:10.1371/journal.pone.0238949)

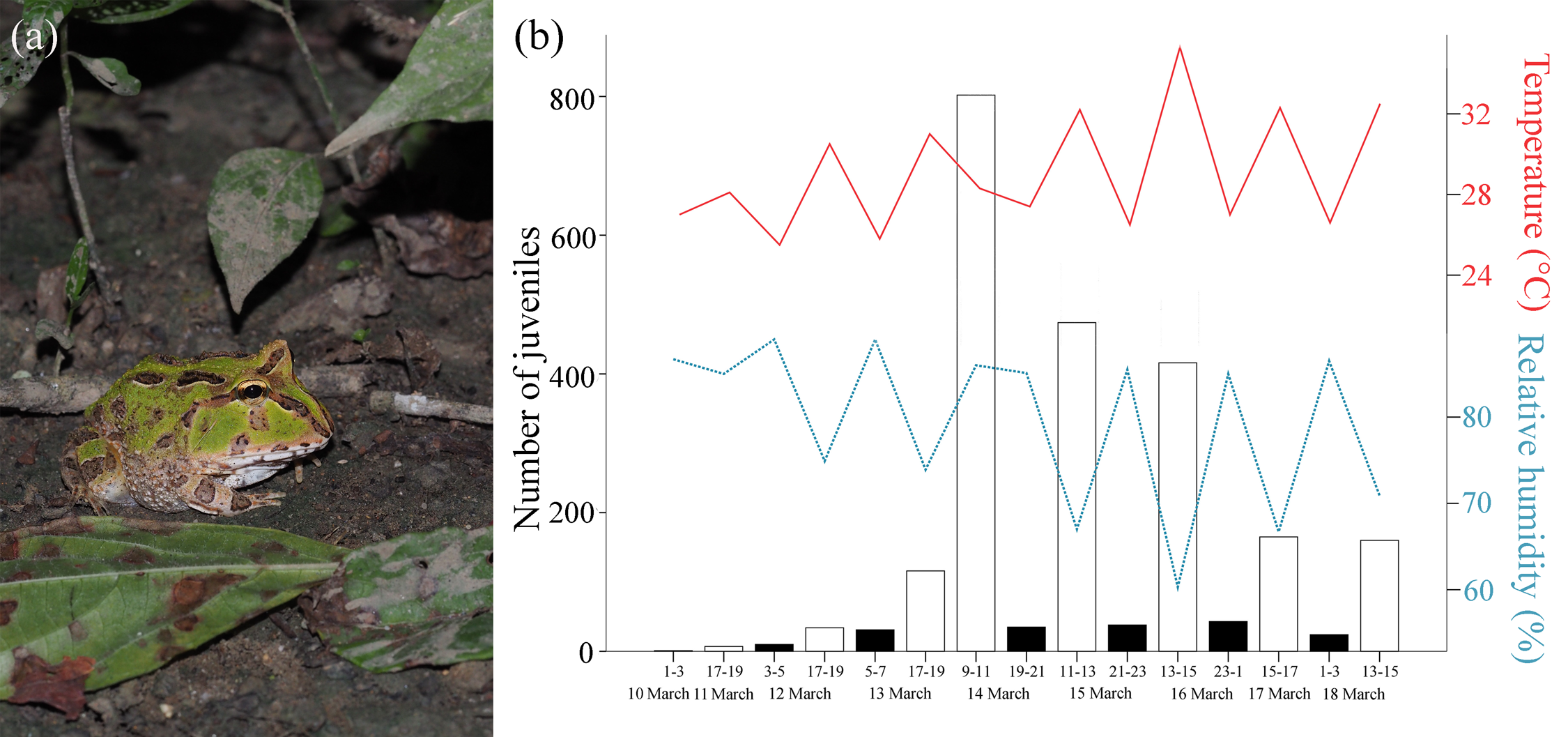

Supplement: S1 Fig — (TIF) [file pone.0238949.s001.tif]

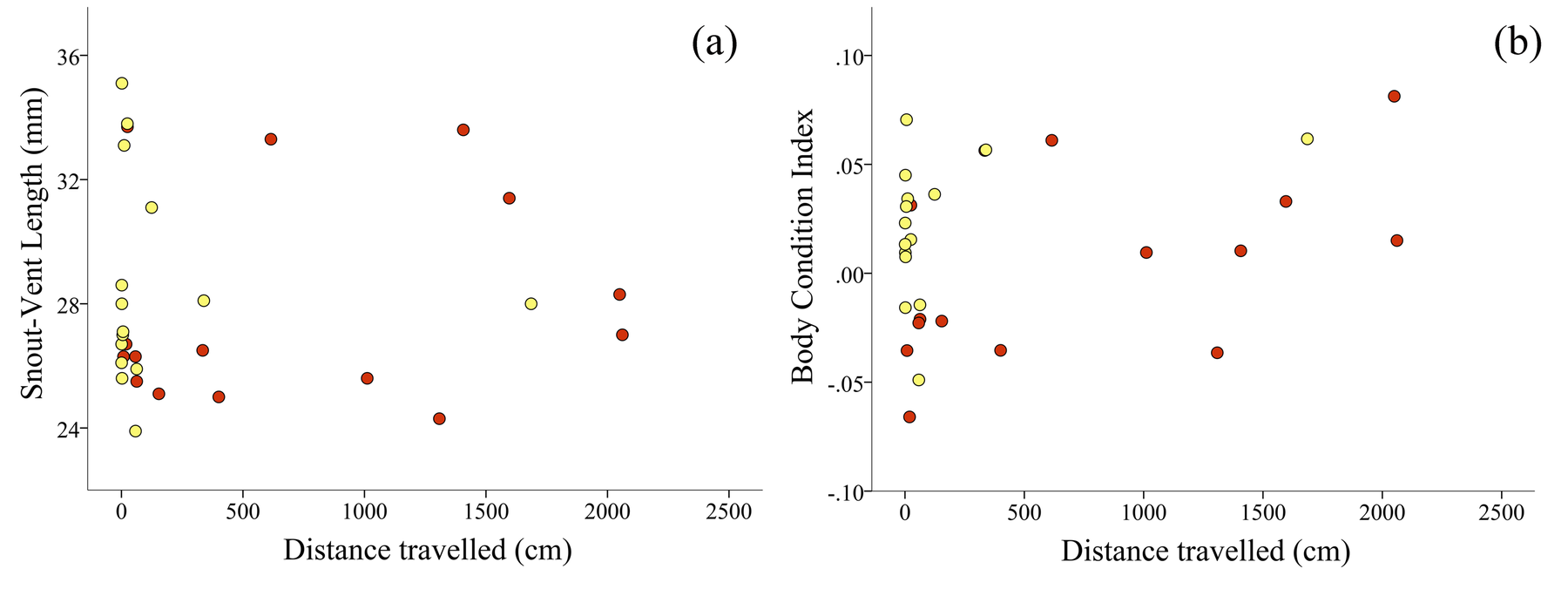

Supplement: S2 Fig — Red dots—single (i.e. no adult), and yellow dots—exposed (i.e. adult visible) treatments, respectively. (TIF) [file pone.0238949.s002.tif]
