# Supplementary material for: Adult—Juvenile interactions and temporal niche partitioning between life-stages in a tropical amphibian
Source: PLoS One. 2020 Sep 14;15(9):e0238949. doi: 10.1371/journal.pone.0238949 (PMC7489520; doi:10.1371/journal.pone.0238949)
Supplement: S1 Table — (DOCX) [file pone.0238949.s003.docx]

**S1 Table.** Schedule of transects and number of encountered Pacific horned frogs, according to their life-stage.

| **Date** | **Start hour** | **No. juveniles** | **No. adults** |
| --- | --- | --- | --- |
| 21 January 2016 | 19:00 | 0 | 10 |
| 22 January 2016 | 21:00 | 0 | 3 |
| 23 January 2016 | 23:00 | 0 | 1 |
| 25 January 2016 | 01:00 | 0 | 7 |
| 26 January 2016 | 03:00 | 0 | 3 |
| 27 January 2016 | 05:00 | 0 | 2 |
| 27 January 2016 | 17.00 | 0 | 0 |
| 28 January 2016 | 19:00 | 0 | 3 |
| 29 January 2016 | 23:00 | 0 | 20 |
| 30 January 2016 | 21:00 | 0 | 15 |
| 01 February 2016 | 01:00 | 0 | 12 |
| 02 February 2016 | 03:00 | 0 | 15 |
| 03 February 2016 | 05:00 | 0 | 11 |
| 03 February 2016 | 17:00 | 0 | 0 |
| 04 February 2016 | 19:00 | 0 | 4 |
| 05 February 2016 | 21:00 | 0 | 21 |
| 06 February 2016 | 23:00 | 0 | 21 |
| 08 February 2016 | 01:00 | 0 | 21 |
| 10 February 2016 | 17:00 | 0 | 0 |
| 11 February 2016 | 19:00 | 0 | 11 |
| 12 February 2016 | 03:00 | 0 | 14 |
| 12 February 2016 | 21:00 | 0 | 6 |
| 13 February 2016 | 23:00 | 0 | 19 |
| 15 February 2016 | 01:00 | 0 | 21 |
| 16 February 2016 | 03:00 | 0 | 11 |
| 18 February 2016 | 05:00 | 0 | 5 |
| 18 February 2016 | 17:00 | 0 | 0 |
| 19 February 2016 | 19:00 | 0 | 1 |
| 20 February 2016 | 21:00 | 0 | 4 |
| 21 February 2016 | 23:00 | 0 | 14 |
| 23 February 2016 | 01:00 | 0 | 2 |
| 24 February 2016 | 03:00 | 0 | 2 |
| 25 February 2016 | 05:00 | 0 | 11 |
| 25 February 2016 | 17:00 | 0 | 0 |
| 26 February 2016 | 19:00 | 0 | 42 |
| 28 February 2016 | 21:00 | 0 | 86 |
| 29 February 2016 | 23:00 | 0 | 45 |
| 02 March 2016 | 01:00 | 0 | 37 |
| 04 March 2016 | 03:00 | 0 | 28 |
| 06 March 2016 | 05:00 | 0 | 19 |
| 06 March 2016 | 17:00 | 0 | 0 |
| 07 March 2016 | 19:00 | 0 | 6 |
| 08 March 2016 | 21:00 | 0 | 8 |
| 09 March 2016 | 23:00 | 0 | 14 |
| 10 March 2016 | 01:00 | 1 | 13 |
| 11 March 2016 | 17:00 | 7 | 0 |
| 12 March 2016 | 03:00 | 10 | 19 |
| 12 March 2016 | 17:00 | 34 | 0 |
| 13 March 2016 | 05:00 | 31 | 3 |
| 13 March 2016 | 17:00 | 116 | 0 |
| 14 March 2016 | 09:00 | 802 | 0 |
| 14 March 2016 | 19:00 | 35 | 3 |
| 15 March 2016 | 11:00 | 474 | 0 |
| 15 March 2016 | 21:00 | 38 | 2 |
| 16 March 2016 | 13:00 | 416 | 0 |
| 16 March 2016 | 23:00 | 43 | 5 |
| 17 March 2016 | 15:00 | 165 | 0 |
| 18 March 2016 | 01:00 | 24 | 1 |
| 18 March 2016 | 13:00 | 160 | 0 |
